# Being between life and death—experiences of COVID-19 survivors 12 to 18 months after being treated in intensive care

**DOI:** 10.1080/17482631.2024.2398223

**Published:** 2024-09-05

**Authors:** Tina Lundberg, Eleonora Falk, Anette Alvariza, Eva Åkerman, Oili Dahl, Marie Nilsson, Lena Anmyr

**Affiliations:** a Medical Unit: Clinical Social Work, Karolinska University Hospital, Stockholm, Sweden; b Department of Health Care Sciences, Marie Cederschiöld University, Stockholm, Sweden; c Department of research and development/Palliative Care, Stockholms Sjukhem, Stockholm, Sweden; d Perioperative Medicine and Intensive Care, Karolinska University Hospital, Stockholm, Sweden; e Department of Health Sciences, Lund University, Lund, Sweden; f Division of Nursing, Department of Neurobiology, Care Sciences and Society, Karolinska Institutet, Stockholm, Sweden; g Academic Primary Care Center, Region Stockholm, Stockholm, Sweden; h Department of Neurobiology, Care Science and Society, Division of Family Medicine and Primary Care, Karolinska Institutet, Stockholm, Sweden; i Department of Clinical Science, Intervention and Technology (CLINTEC), Karolinska Institutet, Stockholm, Sweden

**Keywords:** COVID-19, patients, intensive care, psychosocial wellbeing, psychosocial support

## Abstract

**Purpose:**

This study aims to explore the experiences of care, psychosocial support, and psychosocial wellbeing among patients treated for COVID-19 in intensive care 12 to 18 months after discharge.

**Methods:**

This study used a qualitative approach with a descriptive design. Semi-structured interviews were performed with 20 adult patients treated for COVID-19 12 to 18 months after being discharged from a university hospital in Sweden. Data were analysed using qualitative content analysis.

**Findings:**

The participants were severely affected by COVID-19 both during the hospital stay and afterwards. They experienced overwhelming fears and uncertainties related to their wellbeing and possibility to recover. The care was described chaotic with staff that were stressed; however, the efforts of the staff during this strenuous circumstance were still positively acknowledged. Difficulties to stay in touch with family and friends due to visiting restrictions affected the patient’s psychosocial wellbeing.

**Conclusion:**

Contracting COVID-19 in the beginning of the pandemic was a stressful event. Being seen and heard is of importance as it has the possibility to create a feeling of security and being cared for despite unclarities about treatment and illness trajectory. Accordingly, healthcare staff play an important role for the psychosocial wellbeing of patients treated for COVID-19.

## Introduction

The year of 2020 will go down in history as the start of one of our most strenuous and perilous times. On 11 March 2020, The World Health Organization (WHO) declared the outbreak of COVID-19 as a pandemic (World Health Organization [WHO], 2023). Today almost 7 million people have died of COVID-19 worldwide (WHO, 2023). At the beginning of the pandemic, practically, nothing was known about the disease and its consequences. No effective treatment options were available and at the same time the mortality of the disease became increasingly apparent (Muralidar et al., 2020). In an attempt to slow down the spread of the infection, several countries around the world implemented lock downs or regulations about social distancing (Rajkumar et al., 2022). The mental health impact on patients treated for COVID-19 as well as on the general population has yet to be studied (Torales et al., 2020). It is now known that patients infected by COVID-19 witnessed widespread distress and fear at the threat of a COVID-19 diagnosis and its consequences (Zhang et al., 2022). The mental impact on short but also long-term COVID-19 is extensive (Eklund et al., 2024; Larsson et al., 2023) both on the general population and especially on more vulnerable groups. Various psychological impacts associated with the experience of quarantine have also been reported (Rajkumar et al., 2022).

Sweden was heavily embattled, having the lowest intensive care capacity in Europe with 524 intensive care beds in the country (OECD/European Union, 2022). During the first peak of the COVID-19 pandemic about 750 patients were treated in intensive care units (ICU), of which three out of four were patients treated for COVID-19 (The National Board of Health and Welfare, 2023). This meant that a healthcare system that already lacked registered nurses, especially in emergency care, became even more strained (The National Board of Health and Welfare, 2018). In order to man up the care, healthcare staff were transferred to the ICU-wards, sometimes without proper introduction (Bergman et al., 2021). In addition, the usually flexible visiting regulations changed during the pandemic and most visits were denied except for dying patients (Jensen et al., 2021). This was the situation individuals seriously infected with COVID-19 in Sweden were faced with at the beginning of the pandemic. Studies examining the experiences of care as well as the psychological impact among ICU-patients in the Swedish context during the first months of the pandemic and in the long term are scarce. However, a recent publication emphasizing on facilitators and barriers to recovery among Swedish patients who were cared for in ICU during the COVID-19 pandemic does highlight the fragility of life with persistent difficulties during the rehabilitation process (Eklund et al., 2024). Nonetheless, COVID-19, both by way of its novelty and global historic significance, holds such dignity that further knowledge of the psychosocial consequences for some of those most profoundly affected is not only warranted but essential. It is important to gain more knowledge on how patients with COVID-19 experienced their time in the ICU and how the disease affected their future life in a Swedish context. Examining their psychosocial wellbeing, meaning the personal satisfaction and happiness related to psychological and social factors (Burns & Pachana, 2017), and the psychosocial support, meaning the support of these factors, can bring important insights for upcoming challenges. The knowledge of the patients who were most seriously ill due to COVID-19 and their experiences of care are necessary to inform and prepare the healthcare system for future pandemics. This study aims to explore experiences of care, psychosocial support, and psychosocial wellbeing among patients treated for COVID-19 in intensive care 12 to 18 months after discharge.

## Material & methods

### Design

This study used a qualitative approach with a descriptive design.

The present study is part of an observational longitudinal study: recovery and rehabilitation during and after COVID-19 (ReCOV). The ReCOV-project is a multi-disciplinary collaboration aiming to examine the consequences of COVID-19 from the perspective of patients, next of kin, and healthcare staff (Rydwyk et al., 2021). The present study involves the patients’ experiences of care, psychosocial support, and psychosocial wellbeing.

### Study population and procedure

The inclusion criterion was adult patients suffering from COVID-19 who had been admitted to ICU at the Karolinska University Hospital in Stockholm, Sweden, during the early stages of COVID-19 in the spring of 2020. A purposeful sampling method was used. The participants were chosen to ensure a variation in gender, age, language, hospital site where they had been admitted and time point of admission during the spring of 2020. Language was considered important as a large proportion of those treated for COVID-19 in Sweden during the early stages of the pandemic belonged to underprivileged groups with a high level of non-Swedish speaking population. An interpreter was to be used in interviews with non-Swedish-speaking participants. Participants with varying length of care during the early stages of COVID-19 were selected. The participants were admitted between 25 March 2020, and 29 May 2020. The participants were also selected to avoid that the participants would be approached repeatedly by researchers within the ReCOV-project (Rydwyk et al., 2021). All participants had already consented to participate in research within the ReCOV-project and were sent an information letter which was followed by a telephone call by the interviewers. Six of the primary selected patients declined participation or did not respond and were accordingly replaced with participants with matching characteristics, such as gender and age, as far as possible, until the full study population of 20 participants was reached. In total, 30 patients were approached, of which 10 patients declined or did not respond. Four of the invited patients were non-Swedish speaking, but they all declined participation. Most of the patients did not give any reason for declining or stated that they just did not want to participate, one claimed to have been involved in too many research projects already, one being too tired due to having had a stroke and one having no time.

### Data collection

Data were collected via semi-structured interviews. An interview guide focusing on the experiences of care and psychosocial support as well as the psychosocial wellbeing during and after hospitalization was developed. The guide is included as supplementary material (Supplement online material, 1). The interview guide was piloted; however, no revision of the interview guide was found necessary. An experienced medical social worker or critical care nurse (EÅ, OD) conducted the interviews. The participants were initially given oral information about the study. The sharing of positive as well as negative experiences were emphasized during the interviews. Nineteen interviews were performed using video calls and one by phone as the pandemic did not allow face-to-face interviews. No repeat interviews were carried out, and the transcripts were not returned for comments by the participants. The interviews lasted for approximately 1 hour. The depth and length of the 20 interviews were assessed enough to reach data sufficiency. The time between discharge from hospital and interview varied between 12 and 18 months (*M* = 13).

### Data analysis

The interviews were transcribed by the interviewing medical social worker and a transcriber. The transcripts were then analysed inductively, using qualitative content analysis (Graneheim & Lundman, 2004; Lindgren et al., 2020). A social constructivist stance was taken, acknowledging the researchers' impact on data, however aiming to explore the experiences of COVID-19 from the perspective of participants. The first and the second authors thoroughly read half of the interviews each and extracted meaning units relating to the research questions before they were condensed. The condensed meaning units were then labelled with codes describing the core content. During the analysis process, the first and second authors repeatedly consulted initial transcripts in order to validate the findings. This process was performed in close collaboration between the two authors and in cooperation with the third author to enhance the trustworthiness of the findings. The first and the second author then discussed and developed categories, which were thereafter deliberated in a joint sitting with the remaining authors and with the third author separately. Finally, the first and second authors jointly performed a final validation check by comparing the final results with the original transcripts. Participants did not provide feedback on the findings.

### Ethical considerations

Although the participants had already signed a written consent within the ReCOV-project the information letter that was sent to the participants in the present study emphasizing the voluntariness of participating in the study. This information was also repeated by the researchers in a follow-up telephone call. None of the researchers had any relationship with the participants. To ensure confidentiality, the participant’s identity was replaced with an identification number which was used throughout the study. The data was secured in a safe web-platform (SharePoint) to which only the researchers had access. The ethical principles of the Declaration of Helsinki (World Medical Association, 2013) were followed. Ethical approval was received from the Regional Ethical Review Board in Stockholm (Dnr 2020–02149).

## Findings

The study population consisted of 8 women and 12 men, with a mean age of 56 years, ranging between 26 and 74 years. The length of care was on average 25 days (number of days varied between 8 and 65) (Table I).

**Table I.** Description of participants.

Participant	Age	Gender	Born in	Occupation	Days admitted site	Hospital
P1	26	F	Sweden	Working	15	South
P2	33	M	Sweden	Working	35	South
P3	41	M	Outside Europe	Working	35	North
P4	45	M	Outside Europe	Sick-leave, working 25% or less	27	North
P5	50	F	Sweden	Working	35	North
P6	51	M	Outside Europe	Working	8	North
P7	52	F	Sweden	Working	19	North
P8	54	F	Finland	Working	17	North
P9	56	M	Outside Europe	Sick-leave	47	North
P10	58	M	Outside Europe	Working	21	North
P11	59	M	Outside Europe	Sick-leave	65	North and South
P12	59	F	Outside Europe	Sick-leave, working 25% or less	19	South
P13	61	M	Outside Europe	Sick-leave	39	South
P14	62	F	Sweden	Working	29	South
P15	62	M	Sweden	Retired	40	North
P16	63	M	Finland	Retired, working part time	11	South
P17	66	F	Sweden	Retired, working part time	26	South
P18	67	F	Sweden	Retired	31	North
P19	69	M	Finland	Retired	21	South
P20	74	M	Sweden	Retired, working part time	27	North

The content analysis resulted in three categories: Being between life and death, i.e., relating to the participants state of being between life and death; The meaning of being seen and heard, i.e., relating to the value of being seen and heard and Connecting with normal life, i.e., relating to the striving for normalcy.

### Being between life and death

Participants talked about the time in the hospital as a time characterized by fear and uncertainty whilst being in a state between life and death. They expressed fear of dying, for example, as a result of medical treatments or merely by shutting one’s eyes. Others mentioned being aware of friends and co-patients whose lives had been extinguished by COVID-19 which led to a fear that the same fate awaited them. This fear was sometimes amplified by witnessing the ICU environment. Participants expressed a fear of dying alone without a chance to bid their loved one’s farewell:

[The biggest fear] was that I would simply die, and that the family would not be allowed to come … and say goodbye, sort of. Or if I’m going to lie here alone and die… (P5)

During intensive care, the participants described that they had been in a mixed state of consciousness versus subconsciousness due to heavy sedation, with vague memories or not knowing which was reality. The participants recalled waking up in ICU to feelings of despair or confusion as to where they were and why or how long they had been there. Several accounts were given of horrific hallucinations and nightmares, in some cases leading to beliefs that hospital staff were trying to kill them. Contrastingly, the time at the hospital was also described as a peaceful rest without any frightening memories at all. At times, participants depicted a sense of calm associated with the risk of dying, while others expressed victory and a feeling of having a strong mind while defying impending death. There were also experiences of denial regarding the severity of the illness and that the admission to the ICU was a purely precautionary measure, as exemplified by a participant who was a nurse by occupation:

My thought was that they sent me to the intensive care unit just because I was a nurse, they didn’t want more dead nurses… And I didn’t realize I was that sick, so I thought it was just as a precaution. (P5)

Occasionally, participants described that they were incapable of communicating when they woke up at the ICU, rendering them unable to request answers or alleviate their uncertainty and confusion, which meant having to cope with internal emotional processes alone. Fear of not fully regaining physical or cognitive capabilities were described. The inability to communicate or move, coupled with limited contact with hospital staff and family, was expressed as resulting in an indescribable loneliness.

The participants described the state of between life and death as being focused on the task of survival or simply “breathing”. Being in need of medical equipment in order to breathe fostered scary feelings of lost bodily control, as well as an inability to move, leading to feelings of being incarcerated:

It felt like being trapped, kind of in a prison, a similar feeling I would say. You couldn’t get out of there, there wasn’t much you could do. (P3)

After returning home, participants still suffered from the fears and uncertainties of being between life and death due to COVID-19. Physical as well as psychological symptoms remained, such as shortness of breath, irritability, anxiety, or even regular panic attacks connected to fear of death. Unpleasant flashbacks and nightmares negatively affected the participants’ sleep and physical recovery even after 12 to 18 months. To determine if they had indeed recovered, they wanted to see imaging from scans or test results or requested further testing. Occasionally, participants shared how they had basically become hypochondriacs or antisocial due to an exaggerated fear of illness recurrence. Unexpected and sudden decline at home was connected to a kind of energy depletion resulting in extreme tiredness:

When I came home… my body started shaking… […] And then I slept, I don’t know how long but I woke up the next day, still tired. … It got tougher and tougher with all the impressions and that’s when you sort of understood that you had been in a protected environment and it was tough to process everything, and the fatigue was constant… (P3)

Once home, it had originally been difficult to hear stories or updates concerning COVID-19 but over time, the participants expressed being able to distance themselves. It was considered important for the purpose of healing, to return to normal routines as quickly as possible. This option could, however, be compromised by physical limitations or extreme tiredness that was still ongoing 12 to 18 months after discharge. Feelings of normalcy were furthermore hampered by worry and uncertainty related to an impaired capacity to return to work or fear of economic difficulties as a result of COVID-19.

Participants sometimes described that COVID-19 was the worst thing they had ever experienced, negatively affecting their overall outlook on life. There was a feeling of deteriorated quality of life and no longer recognizing themselves. One participant even expressed disappointment over having survived:

In fact, I would have preferred that I had died instead of survived… When I got home, I was quite disturbed by that. … After having felt this anger that I had survived I had an incredibly bad conscience and [felt] guilt towards the staff who had worked so hard to make me survive and there I sat, ungrateful and thought it was crap that I didn’t die. (P17)

Despite the persisting difficulties, there was a description of an overall physical and emotional improvement over the course of time. In some cases, participants did not feel considerably affected by having had COVID-19, while others emphasized sheer gratitude to be alive, and the victory of conquering death. The experience of COVID-19 was occasionally talked about as a life-changing event. Being so near death had led to a greater appreciation and regard for life, making participants prioritize differently:

You are more ambitious about life, I would say. … you thought you were immortal, like strong … but after something like this, you want to do other things. … There is more to do in life than just sort of work and be the same old person you were before. (P3)

### The meaning of being seen and heard

Participants described the hospital stay as a turbulent time, where the possibility of being seen and heard had often been compromised. Their encounters with healthcare staff were sometimes described as deficient, often due to healthcare staff being stressed or unavailable due to attending to other patients. This could lead to participants feeling like they had to manage on their own. These situations were exemplified by, e.g., being denied help to use the toilet due to the staffs fear of contracting the disease.

There were situations when staff were described as lacking empathyfor example, when participants had to walk in the corridor with their clothes open in the back, worrying about the integrity. Participants also described being ridiculed for hallucinations or met with prejudice due to psychiatric diagnoses. However, sometimes a more direct approach from the staff signalled that they knew what they were talking about which, for some participants, fostered feelings of security. At times, participants described themselves having been scatter-brained or even mean and were thankful that the staff had put up with them. Other times, the participants talked about quarrels with the staff and wished that the staff would have been more understanding, realizing that the patients might not be themselves:

So I had a huge panic because I thought they were going to kill me […]And then I say something awful for sure […] But then he [the staff] says something … little harsh […] You feel like 5 years old and start to cry … saying you’re sorry. I can feel now afterwards that … they should have known that maybe I wasn’t myself, that maybe you should think a little more about what you say to… a patient. (P5)

Sometimes the participants described physical and practical circumstances as troubling. The staff had occasionally been experienced as impersonal or unengaged, with primary focus being placed on physical wellbeing while somewhat neglecting patients’ psychological wellbeing. This led to feelings of not really being seen as a person. Even the physical care was, occasionally, referred to as inadequate, resulting in incorrect medication being administered or injuries being obtained. It was furthermore described as terrifying having to lay beside co-patients who were so ill that the participant could not determine whether the person was dead or alive. There were occasions when participants’ belongings were lost without feeling that staff members cared or understood the consequences or worries it entailed. For example, a lost phone meant isolation:

I [had] not wanted to get rid of my mobile phone of course… because you couldn’t use the ward’s phone due to the risk of infection. (pause) Because that makes you very isolated in many ways. (P14)

At times, participants described that their need for information was neglected. A desire for competent staff were expressed since staff with lower education sometimes were experienced as less empathetic, more speculative and less knowledgeable. Furthermore, some participants expressed a need for more support from different professions. Contrastingly, others described having been offered support but not feeling a need for it. The medical equipment or the staff’s personal protective equipment was not considered a problem when it came to communication possibilities. Communicating using pen and paper or even body language, when they could not speak or hear the staff, was deemed to have worked well, according to participants. One patient explained it was fantastic how *“some staff were capable of smiling with their eyes”*.

Despite the described shortcomings, participants’ overall reflection was that they were satisfied and impressed by the care they had received. A great amount of appreciation was expressed for the staff, realizing their hectic circumstances, particularly while dealing with the challenges of a completely new disease. The staff were often highly praised by the participants, reflecting that they had been seen and heard during their hospital stay. On the whole, the healthcare staff were perceived as positive and doing their best to get to know their patients. The staff were furthermore described as being fantastic, angels, calming, patient, considerate, pushing participants forward, helping them feel secure and not to be afraid. It was appreciated when the staff made sure that the participant got a phone or when they arranged video calls with the patient’s family. Small gestures were specifically mentioned and greatly appreciated, such as being offered an ice-cream or having the hair combed:

My hair was so dirty and… greasy […] and they were combing my hair. […] And then… do you know what they did? Asked which side I usually part my hair on, and they combed it like I usually have it […] I thought oh my god … Really great! (P8)

After returning home and 12 to 18 months later, participants described persisting needs of psychological and physical care as well as rehabilitation. In some cases, participants lacked information on the results of their follow-up examinations and other times they described having to struggle to get the aftercare they felt they needed. Examples were given of delayed rehabilitation or that the primary or psychiatric care did not see or understand their problems. There were descriptions of even being refused psychiatric care despite having suicidal thoughts. Participants were in hindsight trying to piece together the chaotic and confusing time they experienced at ICU and sometimes wished that they would have had a diary to facilitate this process. In other cases, however, participants described satisfaction with the care and support they had received or had been offered from different professionals after returning home. The hospital follow-up was often described as fantastic.

### Connecting with normal life

Participants described that the restraints that followed COVID-19 had led to difficulties having contact with family and friends as hospital visitation was not permitted. At the ICU, it was primarily healthcare staff who handled contact with participants’ families and friends. This was for the most part considered a relief for participants as they lacked the strength to manage these contacts themselves. There were even participants who expressed that they had not cared about their family when they were at the ICU. In contrast, others wished they could have had their families closer during the hospital stay, and they had phoned them as often as possible, as contact with their loved ones gave them strength.

Participant’s possibility to have personal contact with family and friends was described as being facilitated once they were moved to recovery wards. Means of communication were texting, video or regular phone calls, or a device handed by the staff. However, these means of communication were described as difficult to use when participants were too weak to hold a phone or device or when, as in some cases, their phones had been lost during the hospital stay.

The participants described that their family members had gone through a rough time, worrying a lot about them during the hospital stay. According to participants, their families had felt shut out, lacking information on the condition of their loved one. They were described as being left, just sitting and waiting for a call from the hospital. For family members living outside of Sweden, it was hard to get hold of any information at all. Participants described that family members, in some cases, had no way of knowing if the participant was still alive or had died. At other times, family members were informed about participant’s critical condition and that they were allowed to come and bid farewell, which was described as a hard experience for the family members.

They called him [the partner] when … they didn’t know if I would survive… He was allowed to come and put on all the [protective] clothing and enter [my room]. But I wasn’t aware of it, I was sedated at the time. And then he had to leave with my, my engagement ring in this little … denture box, and it was tough for him. (P5)

Once arriving home after being discharged from the hospital, some participants described a feeling of being somewhat unique as a result of what they had been through in relation to COVID-19. As a result, they still found it difficult to return to a normal life 12 to 18 months afterwards. Worries related to what the future would hold were describedfor example, regarding ability to work and possible economic consequences if the participant would not be capable of returning to work or would be denied sickness benefit. A general perception felt was that people in society were often afraid that participants would contaminate them with COVID-19, which in some cases caused participants to feel like outcasts, especially when visiting family in countries with stricter COVID-19 restrictions. Participants expressed it feeling difficult when people around them lacked understanding of what they had gone through and that they 12 to 18 months later were still fighting with the consequences of COVID-19.

People had high expectations and when I couldn’t live up to them, people were like ´but go back [to the hospital]´. But they don’t really understand what you’ve been through. […] Many people like tries to compare, [saying]that I also had covid and so (laughter). But I just keep my mouth shut. … I smile and laugh a little to myself … No one really understands how tough it is. (P3)

Participants described it as tough when they were not able to socialize with friends or workmates as before because of the spread of COVID-19. In addition, concerns could be experienced as hypocritical and dishonestfor example, if the concerns came from family members with whom the participants had a strained relationship. In contrast, other participants experienced support, encouragement, and concern from their families and friends. They described that over time it became easier to talk about what they had gone through with others. Participants also appreciated the concerns from family and friends as it generated feelings of respect and acknowledgement of having gone through something extraordinary. The patients described having received help from family and friends and did not feel that they were treated differently compared to before having contracted COVID-19, despite having to meet outdoors due to COVID-19 restrictions. Spending time with the family was appreciated, specifically the joy of seeing children and grandchildren, was emphasized.

## Discussion

This study describes the experiences of patients who were treated at intensive care due to COVID-19 during the early stages of the pandemic in Sweden. The result shows that the participants were severely affected by COVID-19 both during the hospital stay and 12 to 18 months afterwards, with persisting emotional difficulties. The participant’s psychosocial wellbeing was characterized by overwhelming fears and uncertainties related to their health and possibility of recovery, as well as to uncertainties concerning the care and what to expect of it. During hospitalization, the care was experienced as chaotic with stressed staff, however, the efforts of the staff during the strenuous circumstances were still positively acknowledged making them feel psychosocially supported. Difficulties to stay in touch with family and friends due to visiting restrictions affected the patient´s psychosocial wellbeing.

The participants’ experiences of being between life and death are consistent with previous research. Fear of death, uncertainty, loneliness, hallucinations, and nightmares, as well as remaining psychological and physical symptoms have, as in the present study, been described internationally as well as in Sweden among patients treated for COVID-19 in ICU (Eklund et al., 2024; Kürtüncü et al., 2023; Norouzadeh et al., 2021; Wallin et al., 2022). These symptoms have been shown common also among ICU patients in general (Danielis et al., 2020; Topçu et al., 2017). The levels of death anxiety among patients treated for COVID-19 in emergency care are similar to those admitted to emergency care due to myocardial infection (Çağlar & Kaçer, 2022). However, what set the patients with COVID-19 apart was that COVID-19 was a new disease and knowledge about its prognosis and how to treat it was very much unknown, which is consistent with previous research on COVID-19 (Muralidar et al., 2020). Furthermore, the death anxiety related to COVID-19 was relatively high in the general population as well as in different groups, such as elderly or healthcare staff, as shown in a systematic review and meta-analysis (Özgüç et al., 2021). The constant reports of the mortality of COVID-19 in media and the uncertainty surrounding the disease were intrusive and difficult to defend against (Muralidar et al., 2020), and the fear that marked society during the beginning of the pandemic are probable to have followed the participants into the ICU, which is one way of understanding the prominent fear of death among patients treated for COVID-19 in the present study.

The result furthermore points at factors that were specific in relation to communication during the pandemic. Undoubtedly, a functioning communication is of importance for patients treated for COVID-19 in ICU (Kürtüncü et al., 2023). Family and friends normally play an important role in maintaining the communication between the patient and the staff in ICU (Danielis et al., 2020). A consequence of the visiting restrictions was that the participant’s difficulties to communicate with the staff after waking up at the ICU, as shown in the category *Being between life and death*, could not be alleviated by the help of family or friends. Specific for the pandemic were also the hospital staffs need for protective equipment (Kürtüncü et al., 2023). However, as demonstrated in the category *Being between life and death*, the protective equipment was not perceived a problem or hinder by the participants in their communication with the staff, which concords with the perception of other patients with COVID-19 admitted to ICU in Sweden (Vogel et al., 2023). The results indicate that physical obstacles, such as medical or protective equipment, can be overcome by the efforts of a supportive staff (Kürtüncü et al., 2023). It may also be that the severity of the pandemic in general made the participants more tolerant towards the potential negative effects that protective equipment could have had under other circumstances. It could even be that the participants, who themselves experienced the consequences of COVID-19, if any, were understanding towards the need for protection.

Despite the difficulties related to COVID-19, the participants illustrated in the category *Being between life and death* that COVID-19 had also led to positive effects, which has been demonstrated among patients with COVID-19 internationally as well (Kürtüncü et al., 2023; Sun et al., 2021). In fact, several studies have reported post-traumatic growth after struggling with the trauma inflicted by COVID-19 supporting that the experience can also bring about positive changes in individuals (Adjorlolo et al., 2022; Collazo-Castiñeira et al., 2022; Zhang et al., 2022). It may be argued that the ability to grow could suggest that patients treated for COVID-19 possess some resilience to the event. High levels of resilience as well as post-traumatic growth have been reported among hospitalized COVID-19 survivors (Adjorlolo et al., 2022). However, the relationship between resilience and post-traumatic growth is not clear-cut, which is why it is not possible to draw any firm conclusions about the participants’ resilience without having studied this specifically (Elam & Taku, 2022).

Participants' overall positive perception of healthcare staff can be compared with previous research on patient experiences of ICU care before COVID-19, which found that a positive attitude from healthcare staff makes patients feel better (Topçu et al., 2017). However, in the present study, participants' understanding of the severity of the pandemic may also explain their overall positive feelings towards the healthcare staff, who worked under strenuous conditions, which also has been reported previously among COVID-19 patients (Tsamakis et al., 2021), as they were praised for their efforts in the category *The meaning of being seen and heard*. Despite the described negative experiences of encounters with the staff in the present study, they were perceived as fantastic, doing a very good job. Staff who took the time to do that little extra, exemplified by the participant who got her hair brushed the way she used to have it, were especially appreciated, which is in accordance with other studies examining COVID-19 (Zhang et al., 2022). In international contexts, patients treated for COVID-19 have even explained their gratitude towards the government and their motherland as measures were taken to protect the people (Zhang et al., 2022). However, this experience was contradicted by the participants in the present study who instead described worry about their livelihood and fear of having to fight with the authorities to get their sickness benefits, as revealed in the category *Connecting with normal life*.

As shown in the category *Connecting with normal life*, the visiting restrictions affected the participants negatively as the possibility to gain strength from the presence their loved ones were lost. The importance of family members has been shown in several studies among ICU patients in general as their presence fosters feelings of security, hope, and courage, this despite sedation and vague memories (Danielis et al., 2020; Eriksson et al., 2011; Topçu et al., 2017). Among patients treated for COVID-19 in ICU the absence of family members has been found to foster worry and strain (Norouzadeh et al., 2021). It is also known that flexible visiting policies reduces the frequencies of delirium and anxiety symptoms among ICU-patients (Nassar Junior et al., 2018). However, despite the negative consequences of the visiting restrictions, the present study does at the same time contrast these findings, as some of the participants experienced that the visiting restrictions offered them relief not having to interact with their family when they were preoccupied with themselves. This dual experience, where visiting restrictions were experienced as both negative and positive, has similarly been described among other patients treated for COVID-19 in ICU in Sweden during the beginning of the pandemic (Wallin et al., 2022). This points out the need to gently assess what is best for the patient. As argued by Downar and Kakewich (2021), the needs of the patients and the need for protection have to be carefully balanced. Sometimes the needs of the patient might outweigh the need for protection from the virus, as the quality of care may be compromised with strict visiting restrictions (Downar & Kakewich, 2021; Jensen et al., 2021), and at other times, the visiting restrictions serves both the patient and the need for protection well.

Once being declared healthy and returning home, a fear of re-contracting COVID-19 was exhibited in the category *Connecting with normal life*, which is consistent with previous findings (Eklund et al., 2024; Kürtüncü et al., 2023; Norouzadeh et al., 2021). The pandemic was undoubtedly still ongoing when the participants were dismissed. This feature distinguishes the recovery period of patients treated for COVID-19 in ICU from patients admitted to ICU for other reasons, as they had to deal with the fact that the recovery period was not only an internal challenge but also threatened by external factors, such as not receiving the rehabilitation they felt they needed or not being able to socialize as before due to restrictions or others fears of being contaminated. However, the participants found strength in their recovery by socializing with their family and friends, as also stated in other COVID-19 populations (Norouzadeh et al., 2021). The less extensive societal lockdown in Sweden may thus have facilitated beneficial socializing with others.

Indeed, as shown, the participants' experiences of COVID-19 largely in line with previous research on patients treated for COVID-19. However, patients treated for COVID-19 in international contexts have accounted for experiences of stigma as well as the role of religion that were not found in the present study. A discussion about the absence of these findings will therefore follow in this discussion section. In a systematic review and qualitative meta-synthesis of the psychological experience of COVID-19 feelings of stigma has been displayed. A shame of not having been able to protect oneself from contagion, being interrogated in the contact tracing and being met with discrimination were examples of a stigma (Zhang et al., 2022) that were not described by the Swedish population in the present study. One way of understanding this was that Sweden had a unique way of dealing with the pandemic compared to most other countries in the world. Sweden did implement regulations related to COVID-19, however, to a less extensive degree compared to many other countriesfor example, no lockdown or enforced quarantines were induced (Ludvigsson, 2020). Accordingly, individuals infected by COVID-19 in Sweden lived in a country with societal conditions that differed from large parts of the world, which may explain why experiences of stigma were not accounted for in the present study.

Overall, this study contributes to the existing literature on the effects of COVID-19 on patients treated in ICU in a Swedish context. Psychosocial aspects of healthcare tend to be neglected, particularly when acute medical needs are in the foreground. However, in order to provide optimal and holistic care—even in the face of emergency crisis—it is important to improve the understanding of how psychosocial needs can be met, to which this study makes an important contribution. Participants' descriptions of being between life and death, the value of being seen and heard, and the experience of striving for normalcy inform healthcare about important issues to assess and address. These insights can increase the preparedness for future pandemics.

### Strengths and limitations

A strength of this study is the rich descriptions from participants experiencing COVID-19 in the beginning of the pandemic and 12 to 18 months after they had been cared for in the ICU. The purposeful sampling has allowed participants with different characteristics, which added variety in the result. Despite the fact that all non-Swedish-speaking patients declined participation, the sample came to include participants with another mother tongue than Swedish, which strengthened the results. This study aims to examine the research question in a Swedish context, although the aim of qualitative research is not to generalize the result, the detailed descriptions and the quotes of the participants allows the reader possibility to assess the potential transferability of the result in other populations or contexts. The use of online interviews was beneficial as the participants did not have to put themselves at risk for contagion in a physical interview. Online interviews could also be considered a strength as participants may feel more comfortable and relaxed in their own homes with a distance to the interviewer. Being able to see the interviewer was a benefit. On the other hand, the interviewer could not know if anyone besides the participant were present in the room, which potentially could have affected the interview. Furthermore, online interviews could be limiting if participants felt that this distance hindered them from talking about difficult experiences. However, the interviewers had experience of difficult conversations and conducted the interviews in a calm and open manner to create an atmosphere with the aim of helping the participants to feel secure allowing them to be able to talk anyway.

## Conclusion

Hospitalization due to COVID-19 in the beginning of the pandemic was highly stressful, characterized by fear of death, as much was unknown about treatment and prognosis. Being seen and heard is of importance in order to increase feelings of security and being attended to despite treatment uncertainty and illness trajectory. Accordingly, healthcare staff play an important role in the psychosocial wellbeing of patients treated for COVID-19. Despite being declared healthy, worries about recovery may persist, which suggests that the need for follow-up support should be assessed. This study illustrates the important role healthcare staff have for the psychosocial wellbeing of patients as human interaction has the potential to alleviate suffering.

## Relevance to clinical practice

Despite that COVID-19-vaccine now is available and that it is no longer declared a pandemic, COVID-19 still causes hospitalization and fatality. The results inform healthcare staff about the importance of interaction with patients, which can strengthen their ability to cope during challenging situations and enhance psychosocial wellbeing. Furthermore, it emphasizes the importance of coordinating patients contacts with their family even under challenging circumstances. The results also suggest that the need for follow-up support should be assessed after hospitalization, as COVID-19 related problems may persist even after the infection. In addition, the results may be of importance even in the event of future societal crisis. Future research should elaborate on how the gained knowledge is preserved and kept alive in clinical practice.

## Supplementary Material

Interview guide.docx

Biographical note.docx
