# Supplementary material for: Being between life and death—experiences of COVID-19 survivors 12 to 18 months after being treated in intensive care
Source: Int J Qual Stud Health Well-being. 2024 Sep 5;19(1):2398223. doi: 10.1080/17482631.2024.2398223 (PMC11382733; doi:10.1080/17482631.2024.2398223)
Supplement: Interview guide.docx [file ZQHW_A_2398223_SM5297.docx]

Interview guide patients

**Background questions:**

1. How old are you?
2. Were you born in Sweden? If not, what country were you born in? How long have you lived in Sweden?
3. What is your education? (primary school, upper secondary education, college, university)
4. Do you have an employment? Which?

**During the admission:**

1. What can you tell us about the time that you were admitted at the hospital? How did you experience the hospital stay?
2. How would you describe your mental wellbeing during the hospital stay?
3. How did you experience the encounters with the healthcare staff during the hospital stay? What was experienced as good in the encounters? What did you experience as less good in the encounters?
4. How did you feel that communication worked with the healthcare staff? What worked well in the communication/what worked less well?
5. How did you feel that your needs for emotional support and comfort were met?
6. Would you have wanted any other kind of emotional support and comforting than what you received?
7. To what extent did you have contact with your relatives? How did you have contact? How did you experience the contact? Did you wish the contact would have been different?
8. What was your biggest concern during the hospital stay? Was there any part of the illness trajectory that scared you more?

**After the discharge:**

1. How would you describe your wellbeing? Your mental wellbeing?
2. How did the period when you were infected with Covid-19 affect you emotionally? I your everyday life? In relation to others?
3. Is there any part of your illness trajectory that you have repeatedly re-experienced afterwards in an involuntary and psychologically unpleasant way? For example, having painful memories of the event, having nightmares or feeling like it's happening again?
4. Are there anything you have started doing differently now after the hospital stay or that you avoid completely because it reminds you of the illness trajectory?
5. Do you feel that you have been treated differently after your hospital stay (e.g by family/friends/care workers)?
6. Is there anything else you want to tell us, something important we haven't asked you about?
