# Supplementary material for: Being between life and death—experiences of COVID-19 survivors 12 to 18 months after being treated in intensive care
Source: Int J Qual Stud Health Well-being. 2024 Sep 5;19(1):2398223. doi: 10.1080/17482631.2024.2398223 (PMC11382733; doi:10.1080/17482631.2024.2398223)
Supplement: Biographical note.docx [file ZQHW_A_2398223_SM5296.docx]

**Biographical note**

Tina Lundberg holds a PhD and is a licenced medical social worker. She currently has a postdoctoral position and is director of studies at Marie Cederschiöld University and is also performing research at Karolinska University Hospital. Her are of research is focused on the wellbeing and support of families in end-of-life and COVID-19.

Eleonora Falk is an authorized clinical social worker with a MSc in children and youth sciences and employed at Karolinska University Hospital. Since 2015 Eleonora has primarily worked with people with cystic fibrosis and their families, including conducting research in the field. In the aftermath of COVID-19 she was involved in research projects associated with individual effects of COVID-19 on psychosocial health. Currently, Eleonora is a PhD student working towards a doctorate degree in Healthcare sciences at Marie Cederschiöld University in Stockholm.

Anette Alvariza holds a PhD degree in Caring Sciences and a position as Professor of Palliative Care at Marie Cederschiöld University and Stockholms Sjukhem in Stockholm, Sweden. She is chair of a PhD- programme and also the scientific leader of a Center of Palliative Care Education and Science. She boats a record of almost 100 publications in international scientific journals and her research focuses mainly interventions aiming to support families.

Eva Åkerman is registered nurse and a specialist nurse in intensive care who have been working in intensive care units for many years (Region Skane and Region Stockholm). In 2012 she became a Doctor of Philosophy in Nursing, School of Health Sciences Jönköping University and in 2024 a Docent in nursing, Lund university. Previously she has worked as a manager in research, competence and caring development and education. Today she is active as Docent and university-lecture in nursing at the Institution Care in high technological environments at Lund University. Eva has always been interested of the outcome for patients and relatives after critical illness and ICU care. In Malmö University Hospital she implemented the ICU diary and a follow-up ICU clinic for patients and relatives. Her research interest is recovery after intensive care and what impact the critical illness and intensive care have on patients and relatives physical, psychological, cognitive functions and health-related quality of life.

Oili Dahl is a Registered Nurse, Certified Clinical Nurse, and holds a PhD. She is employed at Karolinska University, and her most recent position was as Head of Nursing Education. Her research focuses on patient recovery after intensive care and major surgery, the significance of leadership for nurses, as well as the impact of the pandemic on nurses' well-being and health. Since June 2021, she has served as the President of the Swedish Nurses Association, showing great dedication to the nursing profession, professional issues, and research.

Marie Nilsson holds a Master's in social work and a PhD from Karolinska Institutet. She works clinically as an oncology social worker at Karolinska University Hospital, as a development manager and teacher at Academic Primary Health Care Center, and researcher at Karolinska Institutet. Her research is within the field of psycho-oncology, cancer rehabilitation, insurance medicine, self-management and e-health innovation. She has also been involved in research concerning sexuality after stroke and has taught sexual medicine at Karolinska Institutet.

Lena Anmyr is currently employed at Karolinska University Hospital at the Department of Women´s Health and Health Professions as section manager for medical social worker. In April 2014, she defended her thesis “Life Circumstances of children and Adolescents after Cochlear Implantation” at the Department of Clinical Science, Intervention and Technology (CLINTEC), Karolinska Institute, Stockholm Sweden. She has since been involved in cross-professional research projects, both nationally and internationally. She has attended several national and international conferences and has published serval research papers focusing on children with hearing loss and deafness. Currently, she is involved in a larger research project ReCOV (recovery and rehabilitation during and after COVID-19) She is responsible for the sub-project on Psychosocial interventions for patients and relatives during hospital care.
